# Automatic or manual arterial path for the ankle-brachial differences pulse wave velocity

**DOI:** 10.1371/journal.pone.0206434

**Published:** 2018-11-01

**Authors:** Juan Francisco Sánchez Muñoz-Torrero, Julián Fernando Calderón-García, Jorge Manuel De Nicolás-Jiménez, Luis García-Ortiz, Enrique Rodilla-Salas, Manuel Angel Gómez-Marcos, Carmen Suárez-Fernandez, Sergio Cordovilla-Guardia, Sergio Rico-Martín

**Affiliations:** 1 Department of Internal Medicine, Hospital San Pedro de Alcántara, Extremadura Health Service, Cáceres, Spain; 2 Iberian Network on Central Hemodynamic and Arterial Structure, Salamanca, Spain; 3 Department of Nursing, School of Nursing and Occupational Therapy, University of Extremadura, Cáceres, Spain; 4 Zona Centro Health Center, Extremadura Health Service, Cáceres, Spain; 5 Biomedical Research Institute of Salamanca (IBSAL), Primary Health Care Research Unit, La Alamedilla Health Center, Castilla León Health Service, Salamanca, Spain; 6 Hospital of Sagunto, University Cardenal Herrera-CEU, CEU Universities, Valencia, Spain; 7 Internal Medicine Department, Hospital Universitario La Princesa, Madrid, Spain; University of Palermo, ITALY

## Abstract

An automated method for measuring arterial path length with devices that determine pulse wave velocity (PWV) in peripheral arteries is frequently applied. We aimed to compare arterial path length measurements based on mathematical height-based formulas with those measured manually and to assess whether the ankle-brachial difference (abD-PWV) measured with the VOPITB device is comparable to that obtained by manual measurements. In 245 patients, a metric measuring tape was used to determine the arterial path length from the suprasternal notch to the midpoint of the VOPITB cuffs wrapped around the extremities, and the results were compared with those obtained with height-based formulas. We examined the relationship between the abD-PWV measured with both methods. The arterial path length measured manually was shorter than that calculated automatically by 5 ± 2 and 30 ± 4 cm—of 13% and 21% for the arms and legs, respectively (difference of 13% and 21%). As a result, the abD-PWV calculated with the automatic method was greater (automatic abD-PWV vs. manual: 462 ± 90 vs. 346 ± 79 cm/s). The Blant Altman plot showed a percentage error of: 15,2%, 7,5% and 17,3% for heart-brachial, heart-ankle length and abD-PWV respectively. In conclusion there were significant differences between manual and automated arterial length measurements and it translates into difference abD-PWV calculate from both methods. However, the Bland-Alman plot showed that abD-PWV was comparable for both techniques. The advantages of height-based formulas for the calculation of arterial path lengths suggest that they may be the recommended method for measuring the abD-PWV.

## Introduction

More than half of cardiovascular events occur in individuals who were previously asymptomatic; therefore, identifying at-risk subjects is of utmost importance for prevention of these events [1]. Generally, the probability of suffering a future cardiovascular event is determined based on the presence of certain risk factors and then quantified by applying region-specific scores [2–4]. However, most events occur in individuals with low or moderate cardiovascular risk [5]; therefore, the predictive value of these factors is modest (c-statistic ≈0.70–0.75) [6]. As a result, other methods to correctly classify individuals based on atherosclerotic arterial damage are being researched [7].

The study of pulse wave velocity (PWV) is recognised by the European Society of Hypertension and the European Society of Cardiology as a useful tool to assess target organ damage [8]. Measurement of the PWV between the carotid and femoral arteries (cf-PWV) is considered the gold standard method for determining aortic stiffness [9]. However, some drawbacks of this method have resulted in the development of other devices that measure the PWV between two points of more accessible peripheral arteries [10]. The latter devices and those that measure central arterial stiffness (cf-PWV) seem shown similar usefulness [11].

The PWV is assessed by dividing the travelled distance by the travel time. The transit time or time delay of the arterial pulse along the arterial pathway can be measured accurately, but distances are usually estimated from measurements obtained from the body surface. The travel distance should also be measured accurately because even small differences may influence the absolute value of the PWV [12,13]. To estimate the carotid-femoral arterial path length, at least nine methods have been described based on measurements using a metric measuring tape on the body surface, and two methods based on body height have been described [14]. Standardisation of the measure of travelled carotid to femoral distance has been widely debated, and an international consensus was required to establish the recommendations for its measurement [9]. However, manual measurement of the arterial path length can be subject to operator errors or to errors related to the anatomy of the patient. To facilitate this process, mathematical height-based formulas have been proposed to estimate the arterial path length when calculating the PWV. Although, height-based formulas overestimate the true arterial path measurements based on the magnetic resonance image [15], generally this method is applied to any device. Moreover the technique is simple, it shortens the examination time and minimises possible measurement errors, compared with manual measurements.

Our group has developed a device (VOPITB) that independently measures the brachial PWV (b-PWV) and the ankle PWV (a-PWV), allowing the comparison of the PWV between both extremities [16]. Since the damage from arteriosclerosis is lower in the arteries of the arms than in the legs, PWV index between limbs provided by VOPITB could be useful. The a-PWV to b-PWV difference (a-PWV minus b-PWV or abD-PWV) correlates with computed tomography (CT) coronary calcium quantification suggesting that they may be of clinical value [17]. In that study, the arterial path length was measured directly from the suprasternal notch to the midpoint of the cuff on the arm and leg. Our hypothesis was that the estimation of arterial path length with a mathematical height-based formula is comparable to that measured manually. The objective of the present study was to evaluate whether the determination of the abD-PWV with the VOPITB device using the arterial path lengths calculated with mathematical formulas is comparable to that measured manually.

## Materials and methods

This is a cross-sectional descriptive study conducted at the Hospital San Pedro de Alcántara, Cáceres (Spain), between April 2014 and May 2016. A total of 245 subjects were recruited. One hundred fifty subjects were referred from the vascular risk clinic at the Department of Internal Medicine, and 95 were recruited among Hospital workers without vascular risk factors, who were selected during their annual medical examination. The study protocol was approved by the local hospital ethical committee (Hospital San Pedro de Alcántara ethical committee; approval number: 18001757), according to the principles of the Declaration of Helsinki. All subjects provided written consent for participation. All patients underwent a medical history and physical examination that included recording age, sex, height, weight and waist circumference. The body mass index (BMI) was calculated according to the following formula: weight (kg)/height^2^ (m^2^). All participants were required to fast for the determination of total cholesterol, HDL-cholesterol, LDL-cholesterol, triglycerides, glucose, and HbA1c values. Using the relevant data, the patients were stratified by vascular risk according to the Framingham tables [2].

### Arterial stiffness measurements

PWV measurement was performed with the VOPITB device according to a previously described technique [16]. Briefly, 4 cuffs were positioned: 2 on the arms, near the elbow flexion crease, and 2 on the legs, close to the ankles. The device performs all functions automatically, including inflating the cuffs and recording pulse waves with an incorporated oscillometric sensor. The PWV is calculated by the distance/time (cm/s) ratio. Time is the transit time from the outlet of the pulse wave from the heart (peak of the R wave in lead I of an electrocardiographic record incorporated in the system) until its arrival at each of the extremities (start of the pulse wave in the cuff record). To determine arterial path lengths, the PWV in each extremity was calculated using two methods: applying mathematical height-based formulas and manual measurements. The automatic formulas were as follows: heart-brachial length (Lhb): Lhb = 0.2195 x height (cm) - 2.0734, and heart-leg length (Lhl): Lhl = 0.8129 x height (cm) + 12.328 [10]. Manual measurements were determined with a non-stretchable metric measuring tape with the subject standing, with legs together and the arms extended at 90 degrees relative to the trunk. The distances from the suprasternal notch to the midpoint of each of the cuffs that were wrapped around the arms and legs were recorded. The PWVs were automatically provided by the VOPITB device in cm/s for each extremity.

### Statistical analyses

Continuous variables are expressed as the mean ± standard deviation, and categorical variables are expressed as percentages. The Kolmogorov-Smirnov test was used to determine whether the variables followed a normal distribution. Continuous variables were compared using Student's t-test or the Mann-Whitney U test, as appropriate, and categorical variables were compared using the chi-square test. Equivalency between arterial path length and abD-PWV measured manually or mathematical height-based formulas was assessed using Bland-Altman analysis. To be considered clinically acceptable, we have used both methods if the percentage error or limits of agreements would have ranged ±20%, as Critchley LA suggested [18].

A value of p < 0.05 was considered statistically significant. Data were analysed with IBM SPSS Statistics version 24 statistical software.

## Results

Of the 245 patients examined, 147 (60%) were males. The mean age of the population was 57 ± 14 years (mean ± SD). The main clinical characteristics by sex are shown in **Table 1**. Differences in weight and height were found between males and females: 85 ± 13 vs. 71 ± 16 kg (p < 0.001) and 170 ± 8 vs. 155 ± 8 cm (p < 0.001), respectively. The abdominal circumference in men compared to women was 102 ± 12 vs. 95 ± 15 cm (p < 0.001). Males, compared with females, had a higher 10-year cardiovascular risk of presenting ischaemic heart disease according to the Framingham scale, 46% vs. 17%; (p < 0.001). No significant differences were found in the other variables studied.

**Table 1:** Clinical characteristics of subjects included in study.

	All (n = 245)	Women (n = 98)	Men (n = 147)	p
**Age (years)**	57 ± 14	59 ± 15	56 ± 13	0,069
**Weight (Kg)**	79 ± 16	71 ± 16	85 ± 13	<0,001*
**Height (cm)**	164 ± 11	155 ± 8	170 ± 8	<0,001*
**BMI (kg/m**^**2**^**)**	29 ± 5	29 ± 7	29 ± 4	0,505
**WC (cm)**	99 ± 13	95 ± 15	102 ± 12	<0,001*
**Framingham Risk Score**				
Low, n (%)	89 (36)	49 (50)	40 (27)	<0,001*
Moderate, n (%)	71 (29)	32 (33)	39 (26)	0,301
High, n (%)	85 (35)	17 (17)	68 (46)	<0,001*

BMI: Body mass index; WC: Waist circumference Data are mean ± SD

*p<0,05: significant differences between men and women.

**Table 2** shows the differences between manual measurement of arterial path lengths and height-based formulas and the abD-PWV resulting from the measurement of the arterial path length with each method. The difference of the arterial path length measured manually from that calculated automatically was shorter for the arm than for the leg: 5 ± 2 cm and 30 ± 4 cm, respectively. Manual measurements resulted in 13% and 21% lower than the height-based values for the arm and leg, respectively, and these values were similar for both men and women.

**Table 2:** Differences between arterial path length and abD-PWV with manual measurement compared against height-based formulas.

		All	Women	Men	P value*
Heart-Brachial (cm)	Manual	29 ± 4	27 ± 3	31 ± 3	< 0,001
	Heigth-based formulas	34 ± 2	32 ± 2	35 ± 2	< 0,001
	Differences (%)	5 ± 2 (13)^#^	5 ± 2 (16)^#^	4 ± 2 (12)^#^	0,403
Heart-Ankle (cm)	Manual	116 ± 8	110 ± 6	120 ± 6	< 0,001
	Heigth-based formulas	146 ± 9	138 ± 6	151 ± 6	< 0,001
	Differences (%)	30 ± 4 (21)^#^	28 ± 4 (21)^#^	31 ± 4 (21)^#^	0,958

*Differences between men and women.

^#^p <0,001 for manual vs height-based formulas.

abD-PWV: ankle brachial Difference Pulse Wave Velocity.

**Fig 1:**
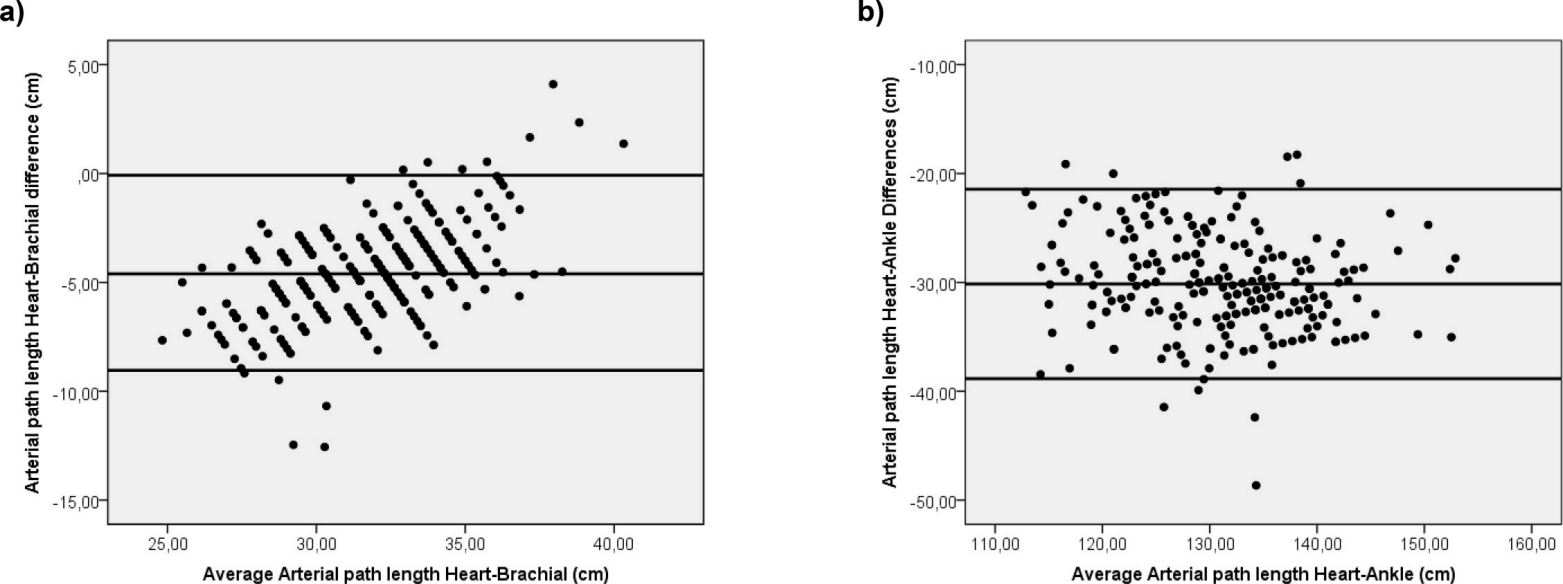
**Bland Altman plots for manual measured and automatic estimated arterial path length for heart-brachial (a) and heart-ankle (b)**.

**Fig 2:**
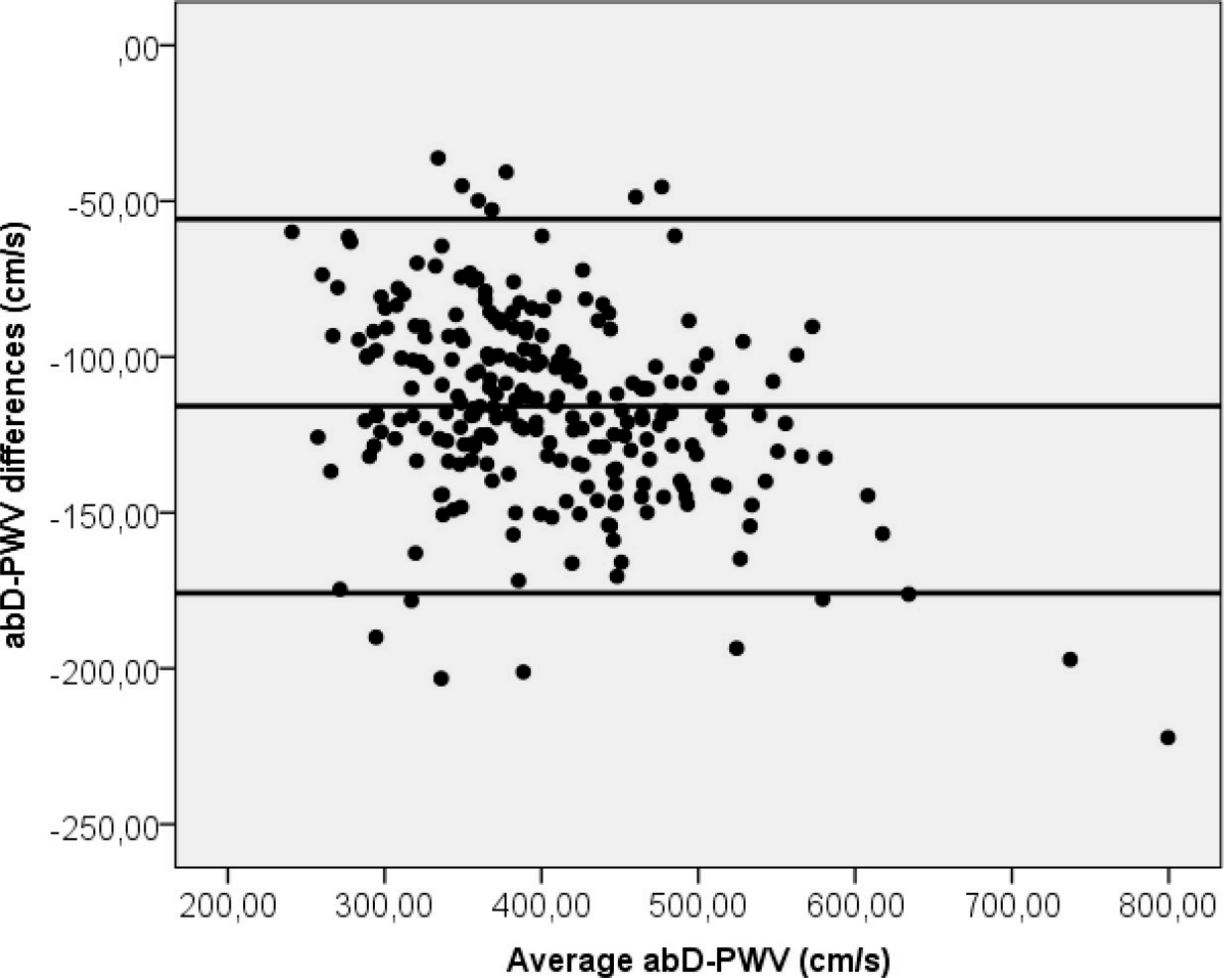
Bland-Altman plot for abD-PWV measured with height-based formulas and manual.

**Fig 1** displays Bland-Altman plots for manual measured and automatic estimated arterial path length for heart-brachial and heart-ankle. A mean differences between the two measure was 5 cm with the limits of agreement from (0,08–9,04) for heart-brachial length and 30 cm (21,44–38,91) for heart-ankle length. The percentage error was 15,2% and 7,5% respectively. **Fig 2** shows Bland-Altman plots for abD-PWV measured with height-based formulas and manual. A mean differences between the two methods was 345,9 cm/s with the limits of agreement from (190,6–501,2). The percentage error was 17,3%.

## Discussion

In the present study, we found that arterial path length estimation from the heart to the arm and ankle is higher when calculated with mathematical height-based formulas than when measured manually. The differences between the two methods were proportionally higher for the arterial path in the leg than in the arm. As a result, the abD-PWV was greater when using arterial path lengths obtained with the proposed formulas than with those measured manually. However, the Bland-Altman plot showed that abD-PWV was comparable for both techniques, suggesting that both can be used to calculate the abD-PWV without evidence to favour any technique because true arterial path was not studied.

The most suitable method for arterial path length estimation for the calculation of the PWV has been widely debated [19]. Depending on the technique used, differences in measurements may affect the calculation of the PWV [13,14]. To establish the gold standard test of arterial stiffness—the aortic PWV (cf-PWV)—an international consensus was required to standardise the method of arterial path length estimation [9]. Devices that measure the PWV in the peripheral arteries, such as the Omron VP-1000 and Vasera, avoid manual measurements by automatically calculating the arterial path length [20, 21]. However, to our knowledge, no validation studies of the method used have been reported for any of these devices.

Our group has developed a device—VOPITB (—that operator independent measures the PWV in the arm and leg, allowing a comparison of the PWV measured in both extremities. Although the abD-PWV, theoretically may be a less ideal measure of large artery stiffness than aortic PWV, since it is a hybrid measure that integrates the mechanical properties from both the central and peripheral arteries, the utility of VOPITB has been demonstrated [16]. The difference in the PWV between the leg and the arm (abD-PWV) has been associated with CT coronary calcium quantification in subjects without symptomatic arterial disease, suggesting that the abD-PWV could be clinically useful in vascular risk stratification [17]. Arterial path length estimations, used to calculate the abD-PWV with the VOPITB device, were performed manually by measuring the distance from the suprasternal notch to the midpoint of the cuffs wrapped around the extremities (heart-brachial and heart-ankle). Because this was a research study, the process was meticulously executed to minimise possible errors. However, if the use of the VOPITB device were to be generalised and introduced in clinical daily practice, a greater likelihood exists of making measurement errors or mistakes when inputting data into the device. In addition, the exploration time increases when arterial path lengths are measured manually, and this could be an added disadvantage.

In the present study, we observed that arterial path lengths calculated with height-based formulas are longer than those measured manually. This difference was greater for the arterial path to the ankle than that to the arm and was directly related to the longer length of the arterial path to the ankle. The distance between the suprasternal notch—the reference point for manual measurements—and the anatomical location of the aortic valve—the reference point for the automatic formulas—could explain the differences found between the arterial path lengths calculated with the two methods. Because the formulas increase the distance to the ankle more than that to the arm, the a-PWV is proportionally higher than the b-PWV; therefore, the difference between the two—the abD-PWV—is greater when applying the mathematical formulas than that obtained with manual measurements. Despite these discrepancies, the percentage error for heart-limb and abD-PWV calculated with Bland-Altman analysis were less 20%, suggesting that two techniques were equivalents [18]. Therefore, the calculation of the abD-PWV could be performed with either method, provided the comparisons of the abD-PWV are made with the same technique. The association was weaker in males, possibly due to differences in body structure. Men tend to have greater abdominal circumference with age, resulting in a greater heart-ankle distance when measured manually than that calculated automatically. However, the actual influence seems negligible in this study.

The height-based formulas evaluated in this study were validated in an Asian population by Sugawara et al [15]. Manual distance measurements were compared to the actual arterial pattern by three-dimensional magnetic resonance imaging. The relationships between the distances from the suprasternal notch to the ankle or arm showed an excellent correlation with the height of the subject [22]. Because the length of the legs in Eastern people is shorter than in other races, it is reasonable to raise doubts about the usefulness of these formulas in other regions [23, 24]. The results of our research suggest that the arterial path estimates with these formulas developed for Asians can also be applied to Mediterranean populations.

Our study has some limitations that should be considered. We did not find studies comparing length measurement for PWV using different methods. We have not found any studies comparing length measurements for peripheral PWV using different methods. Therefore, an accuracy of percentage error less than ±20%, suggested by other cardiovascular study [18], was considered. All patients belong to the same centre in southern Europe, with different anthropometric characteristics from the inhabitants of other latitudes; therefore, these results should be confirmed in other populations. In addition, a decrease in height occurs in many women with age due to osteoporosis of the spine; therefore, the effect of height on the abD-PWV should also be investigated [25]. The VOPITB PWV measures in limbs contains also the aorta and iliac arteries, thus although peripheral arteries are not strictly assessed, the arterial path analysed is longer and may provide more information about arterial disease.

## Conclusions

In conclusion, the present study shows that the calculation of the abD-PWV with the VOPITB device using the arterial path lengths calculated with height-based formulas is different to that obtained with manual measurements. However, the Blant-Altman plot showed that percentage error for abD-PWV was comparable for both techniques. The advantages of using these formulas—including a shorter exploration time and reduced measurement errors—make it the recommended method for the calculation of the abD-PWV in clinical daily practice.

## Supporting information

S1 File Supplemental material.docx. (DOCX)
