# Supplementary material for: Automatic or manual arterial path for the ankle-brachial differences pulse wave velocity
Source: PLoS One. 2018 Nov 1;13(11):e0206434. doi: 10.1371/journal.pone.0206434 (PMC6211696; doi:10.1371/journal.pone.0206434)
Supplement: S1 File — (DOCX) [file pone.0206434.s001.docx]

**S1 Table.** Clinical characteristics of subjects included in study (Suplemental material)

|  | **All**  (n=245) | **Women**  (n=98) | **Men**  (n=147) | **p** |
| --- | --- | --- | --- | --- |
| **SBP (mmHg)** | 133 ± 16 | 131 ± 18 | 134 ± 15 | 0,222 |
| **DBP (mmHg)** | 77 ± 10 | 76 ± 10 | 79 ± 10 | 0,003* |
| **PP (mmHg)** | 55 ± 16 | 55 ± 16 | 55 ± 13 | 0,813 |
| **Glucose (mg/dL)** | 108 ± 37 | 111 ± 48 | 107 ± 27 | 0,454 |
| **Hb1Ac (%)** | 6,3 ± 1,2 | 6,4 ± 1,4 | 6,2 ± 1,0 | 0.223 |
| **Creatinine (mg/dL)** | 0,92 ± 0,24 | 0,80 ± 0,25 | 1,00 ± 0,19 | <0,001* |
| **Total cholesterol (mg/dL)** | 191 ± 42 | 199 ± 45 | 185 ± 39 | 0,010* |
| **HDL-cholesterol (mg/dL)** | 54 ± 15 | 63 ± 17 | 48 ± 11 | <0,001* |
| **LDL-cholesterol (mg/dL)** | 111 ± 39 | 116 ± 42 | 109 ± 36 | 0,164 |
| **Triglycerides (mg/dL)** | 127 ± 70 | 111 ± 60 | 139 ± 74 | <0,001* |
| **Carotid ultrasonography** |  |  |  |  |
| IMT (mm) | 0,74 ± 0,16 | 0,70 ± 0,15 | 0,77 ± 0,17 | 0,002* |
| Presence carotid plaques, n (%) | 134 (55) | 46 (48) | 88 (61) | 0,051 |
| **ABI** |  |  |  |  |
| Left-ABI | 1,11 ± 0,15 | 1,11 ± 0,15 | 1,12 ± 0,15 | 0,173 |
| Right-ABI | 1,10 ± 0,19 | 1,10 ± 0.17 | 1,10 ± 0,21 | 0,291 |

Data are mean ± SD; *p<0,05: significant differences between men and women.

SBP: Systolic blood pressure; DBP: Diastolic blood pressure; PP: Pulse pressure; HbA1C: Hemoglobin glycated; IMT: Intima-media thickness; ABI: Ankle-brachial Index.
